# Transcriptional response to interferon beta-1a treatment in patients with secondary progressive multiple sclerosis

**DOI:** 10.1186/s12883-015-0495-x

**Published:** 2015-11-21

**Authors:** Michael Gurevich, Gadi Miron, Rina Zilkha Falb, David Magalashvili, Mark Dolev, Yael Stern, Anat Achiron

**Affiliations:** Multiple Sclerosis Center, Sheba Medical Center, Ramat-Gan, Israel; Sackler School of Medicine, Tel Aviv University, Tel Aviv, Israel

**Keywords:** Multiple sclerosis, Secondary progressive, Interferon beta, Gene-expression

## Abstract

**Background:**

Interferon (IFN) beta-1a is an approved treatment for relapsing remitting multiple sclerosis (RRMS) and has been examined for use in secondary progressive multiple sclerosis (SPMS). However, no information regarding blood transcriptional changes induced by IFN treatment in SPMS patients is available. Our aim was to identify a subgroup of SPMS patients presenting a gene expression signature similar to that of RRMS patients who are clinical responders to IFN treatment.

**Methods:**

SPMS patients (*n* = 50, 20 IFN treated and 30 untreated) were classified using unsupervised hierarchical clustering according to IFN inducible gene expression profile identified in RRMS clinical responders to treatment. IFN inducible gene expression profile was determined by finding differentially expressed genes (DEGs) between IFN treated (*n* = 10) and untreated (*n* = 25) RRMS patients. Validation was performed on an additional independent group of 27 SPMS IFN treated patients by qRT-PCR.

**Results:**

One hundred and four DEGs, enriched by IFN signaling pathway (*p* = 7.4E-08), were identified in IFN treated RRMS patients. Classification of SPMS patients based on these DEGs yielded two patient groups: (1) IFN transcriptional responders (*n* = 12, 60 % of SPMS treated patients) showing gene-expression profile similar to IFN treated RRMS patients; (2) IFN transcriptional non-responders (*n* = 8) showing expression profile similar to untreated patients. IFN transcriptional responders were characterized by a more active disease, as defined by higher EDSS progression and annual relapse rate.

**Conclusion:**

Within the IFN treated SPMS population, 60 % of patients have a transcriptional response to IFN which is similar to that of RRMS patients who are IFN responders to treatment.

**Electronic supplementary material:**

The online version of this article (doi:10.1186/s12883-015-0495-x) contains supplementary material, which is available to authorized users.

## Background

Secondary progressive (SP) multiple sclerosis (MS) is the progressive phase of relapsing remitting (RR) disease characterized by a shift to continuous accumulation of neurological damage, with or without occasional relapses or minor remissions [1].

As MS progresses from the RR to SP stage, a shift also occurs in the underlying disease pathogenesis. This shift reflects a change from a predominantly autoimmune disease process to one with both inflammatory and neurodegenerative elements [2]. Neurodegenerative components have been demonstrated in pathological and imaging studies by documenting axonal injury in areas devoid of inflammatory response including normal appearing white and grey matter, as well as generalized brain atrophy [3, 4]. The increased importance of neurodegeneration in SP is one possible explanation as to why some disease modifying immunomodulatory therapies (IMD), which are effective in RRMS, lose their efficacy later on [5]. However, evidence derived from pathological examination of brains has shown that neurodegenerative processes are dependent on an ongoing inflammatory activity as demonstrated by coexistence of neurodegeneration and inflammatory infiltrates, including active T and B cells [6].

Currently, interferon (IFN) beta-1b is the only immunomodulatory treatment approved for use in SPMS patients. However, the two main clinical trials, the European [7] and North American [8] trials, which examined the efficacy of this treatment, have shown divergent results [7–9]. The European trial has shown IFN beta-1b delayed progression of disability whereas in the American trial this finding was not replicated. These contradictory results have subsequently been attributed to differing clinical characteristics of patients enrolled in trials, with better treatment outcome associated with patients at an earlier, more active stage of disease as demonstrated by shorter disease duration and higher relapse rate prior to trial initiation [9]. As there is a heterogeneous clinical response to treatment in SPMS, it is important to discover biomarkers which can help identify those patients who could benefit most from therapy.

Gene expression of immunocompetent cells provides a direct representation of disease mechanisms in play. Whereas treatment effects, seen clinically or on MRI, take a long time to evolve, changes on the molecular level are evident much sooner [10, 11]. The gene expression effects of IFN treatment have been well characterized in immunocompetent cells obtained from RRMS patients [11, 12]. These effects, consistently and repeatedly replicated, have become widely accepted as the "IFN signature" [13, 14]. Although the precise mechanism, by which IFN treatment exerts benefit in RRMS, is unknown, this "IFN signature" affects biological pathways related to antiviral activity, immune-regulation, cell survival and apoptosis, cell cycle control, and transcription regulation [15]. Specific interferon inducible genes have been identified that correlate with a better clinical response to IFN treatment in RRMS patients [16, 17], in addition it has been shown that pre-treatment baseline levels of IFN- inducible genes are predictive of clinical response to treatment - with a low expression of IFN induced genes prior to treatment associated with better clinical response [18, 19]. In SPMS, transcriptional studies are much sparser than in RRMS. Studies have examined differences in signatures between non treated SPMS compared to RRMS patients, and have shown minor differences between MS subgroups [20]. However, no transcriptomic studies, regarding blood transcriptional changes induced by IFN treatment in SPMS patients, are currently available.

We suggest that within the SPMS population some patients have RRMS like inflammatory components targeted by IFN. Therefore, the objective of the current study was to find a subgroup of IFN treated SPMS patients, presenting a gene expression signature similar to that of clinical responders to IFN treated RRMS patients. This information could be used to assist in clinical decision making regarding continuation of IFN treatment in RRMS patients who are progressing to the SPMS disease pattern.

## Methods

### Study design

This study is a retrospective exploratory study in which interferon treatment induced gene expression signatures in clinically responding relapsing remitting MS patients were used to classify secondary progressive multiple sclerosis patients undergoing IFN treatment. Peripheral blood mononuclear cells (PBMC) were collected from RRMS and SPMS patients. Demographic and clinical characteristics of patients are presented in Table 1. All patients were diagnosed according to McDonald's 2010 diagnostic criteria [21].

**Table 1:** Demographical and clinical characteristics of patients

Groups	RRMS	SPMS
*N*	35	50
Mean age (years)*	40.2 ± 1.2	52.2 ± 1.1
F (M)	20 (15)	34 (16)
IFN treated (untreated)	10 (25)	20 (30)
Mean EDSS*	2.2 ± 0.2	6.8 ± 0.1
Mean disease duration (years)*	6.8 ± 0.8	18.1 ± 1.4

*p-value <0.05

First, in order to obtain a gene expression profile associated with good clinical response to treatment, an IFN inducible transcriptional profile was determined. This was achieved by identifying differentially expressed genes (DEGs), between clinically responsive IFN treated and untreated RRMS patients. We specifically chose to compare RRMS patients who were clinically responsive to IFN treatment, with RRMS patients who were untreated. This approach, unlike comparing treatment responders vs. non-responders, enabled us to evaluate the characteristic signature of changes in gene expression profile, which were triggered by exposure to interferon in the responding patients.

Inclusion criteria for RRMS patients were: (a) Extended disability status score (EDSS) < = 4.5; (b) IFN treatment for at least 4 months prior to study initiation; (c) Good response to IFN treatment, defined as no acute relapse and no progression in disability during the following 2 years of treatment.

**Fig. 1:**
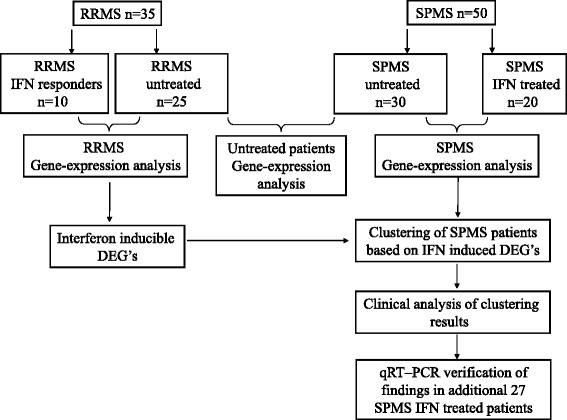
Study design

Next, SPMS patients were classified, using unsupervised hierarchal clustering, based on RRMS IFN induced transcriptional profile. Verification of findings was performed on an additional independent group of IFN treated SPMS patients. Inclusion criteria for SPMS patients were: (a) EDSS > = 5.0; (b) EDSS progression by at least 1 point during the 2 years preceding the study; (c) IFN treatment for at least 2 years prior to study initiation. Study design is shown in Fig. 1.

All patients were steroid free and untreated patients were also IMD treatment free for at least 30 days prior to blood sampling. Treated patients received IFN-beta-1a (Rebif new formulation Merck-Serono), at a dose of 22 or 44 mcg, administered subcutaneously 3 times per week.

### Microarray preparation

Total RNA from PBMC was extracted using Trizol (Invitrogen, USA) and Phase-Look-Gel columns (Eppendorf, Germany). RNA quality was determined by BioRad Experion automatic electrophoresis station. cDNA was synthesized using the One-Cycle cDNA Synthesis Kit, and transcribed by GeneChip IVT Labeling Kit (Affymetrix, Inc. CA.), and hybridized to HGU133A or HGU133A-2 microarrays, washed in a GeneChip Fluidics Station 450 and scanned on GeneArray-TM scanner (G2500A, Hewlett Packard) according to Affymetrix Inc protocol.

### Data pretreatment, normalization and statistical analysis

Microarray data was normalized by R Bioconductor Packages [22] as follows: a) arrays were normalized using single-sample microarray normalization [23]; b) Batch effect was treated by Combat SVA package [24]. Partek Genomics Software was used for statistical analysis. Differentially expressed genes (DEGs) were determined using *p*-value cut off <0.05 after Bonferroni correction for multiple comparisons.

DEG's were applied for functional analysis using QIAGEN’s Ingenuity Pathway Analysis (IPA®, QIAGEN Redwood City, www.qiagen.com/ingenuity). Significantly enriched pathways passed threshold of *p* < 0.05 after Benjamini-Hochberg correction. Classification of SPMS patients was done by unsupervised hierarchical clustering using Euclidean dissimilarity and average linkage algorithm. All continuous parameters including patient clinical and demographical characteristics are presented by mean ± standard error.

### IFN neutralizing antibody (NAB) testing

Quantifying of NAB, in serum samples of IFN treated SPMS patients, was performed after two years of treatment according to a method described by Bertolotto et al. [25].

### Verification of key IFN inducible genes

cDNA was prepared using 1 μg of the total RNA as template with AMV Reverse Transcription System (Promega, USA). In order to determine mRNA expression, real-time quantitative reverse-transcription-PCR (qRT-PCR) was performed using TaqMan technology (Applied Biosystems, USA). HPRT1 mRNA levels were measured as internal control to normalize for mRNA input. The differences between patient groups were assessed using T – test statistic. Significance level was defined as *p* < 0.05.

### Ethics, consent and permissions

This study was approved by the Sheba Medical Center Institutional Review and Ethical Board; all patients gave written informed consent.

### Availability of supporting data

The data set supporting the results of this article, including all raw and processed microarray data and patient parameter metadata, is available in the GEO omnibus, accession number GSE73608.

## Results

### IFN inducible transcriptional profile associated with good clinical response to treatment in RRMS patients

IFN treated RRMS patients with good clinical response to treatment (*n* = 10, mean age 40.9 ± 2.3 years, 4 females, disease duration 5.9 ± 1.5 years, EDSS 1.6 ± 0.4), were compared to untreated RRMS patients (*n* = 25, age 38.1 ± 1.0 years, 16 females, disease duration 7.2 ± 1.1 years, EDSS 2.4 ± 0.2). No statistically significant differences existed between untreated and treated RRMS patients in relation to age, gender, EDSS and disease duration at time of blood sampling. IFN treated RRMS patients exhibited no disease progression, with a negative average change in EDSS of −0.3 ± 0.2 points and no relapses after two years of treatment.

**Fig. 2:**
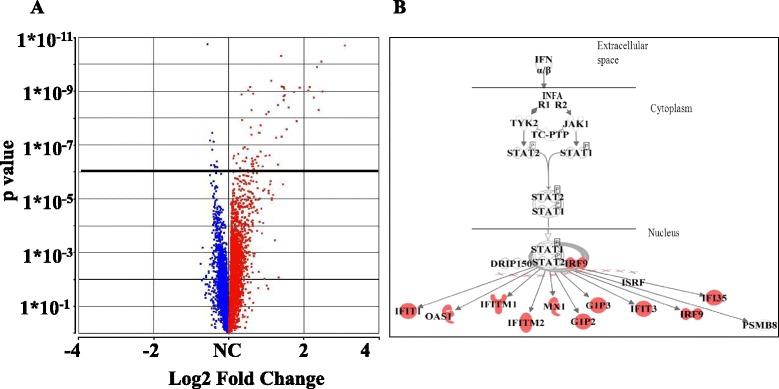
Differential gene expression of RRMS IFN responders compared to untreated RRMS. **a**. Volcano plot - Each dot represents a gene, red dots demonstrate positive fold change and blue negative fold change. Cut off line showing *p*-value level of 0.05 by Bonferroni multiple correction, with 104 DEG’s above cut off line. **b**. Enrichment of IFN signaling pathway within RRMS IFN responders. Up-regulated genes are marked in red

Differential gene expression analysis yielded 104 DEG's, of which 87 were over-expressed and 17 under-expressed (Fig. 2a). A complete list of DEG’s is available in Additional file 1: Table S1. Examining enrichment of these DEGs, using Ingenuity database, showed high enrichment level for IFN type 1 pathway (*p* = 1.7E-16), specifically for IFN beta signaling (*p* = 7.4E-08). According to Ingenuity database, 50 of 104 DEGs are known interferon inducible genes. Amongst these genes, those exhibiting the greatest fold change (2.9-1.8 log2 fold change) include IFN inducible genes such as: IFI44L, IFIT1, IFI44, IFIT3, MX1, ISG15, SIGLEC1, OAS3, and OAS1 genes. Figure 2b demonstrates key DEGs involved in the canonical IFN beta signaling pathway.

### Transcriptional profile distinguishing between untreated SPMS and RRMS patients

SPMS untreated patients (*n* = 30, mean age 51.3 ± 1.4 years, 17 females, disease duration 19.5 ± 1.4 years, EDSS 7.1 ± 0.1) were compared to RRMS untreated patients. We found 147 DEGs, of which 131 were under expressed and 16 were over expressed among SPMS patients. A complete list of DEGs is available in Additional file 2: Table S2. These DEGs were characterized by enrichment of genes which are associated with suppression of inflammatory processes in SPMS patients, as demonstrated by decreased immune response of leukocytes (*p*-value 1.3E-02), and decreased cell proliferation (*p* = 2.7E-04). There was a decreased expression of pro-inflammatory genes, which are well-known MS associated genes, such as: CCL2, IL-12RB1, and IL-23. In addition, canonical MS related IL-17 pathways, were found to be suppressed (*p* = 1.4E-03) with genes such as: CCL2, MAPK11, MUC5B, TRAF3IP2 (being under expressed).

### Classification of SPMS patients based on IFN induced DEGs of RRMS clinical responders

**Fig. 3:**
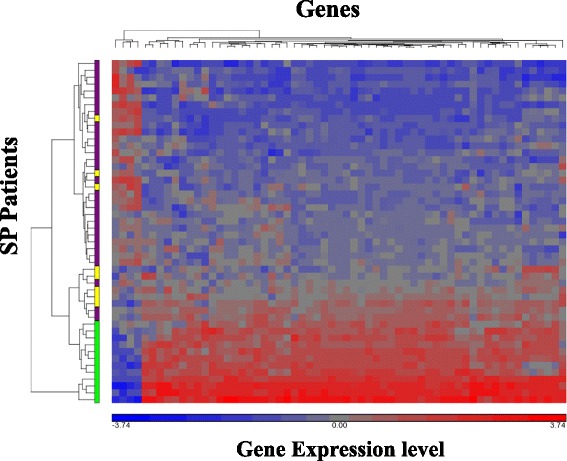
Clustering of SPMS patients based on 104 DEGs. Heatmap depicting hierarchical clustering of SPMS patients according to expression of 104 DEGs, upregulated genes demonstrated in red and down regulated in blue. The left vertical panel presents treatment status of SPMS patients: non treated -purple, IFN treated transcriptional responders- green, IFN treated transcriptional non-responders - yellow

Unsupervised hierarchical clustering of SPMS patients, based on the 104 DEGs associated with IFN treated RRMS patients, yielded two distinct patients clusters (Fig. 3). One cluster, defined as ‘SPMS IFN transcriptional responders’, consisted of 12 IFN treated patients comprising 60 % of SPMS treated cohort. These SPMS patients showed a similar transcriptional response profile to that seen in the RRMS IFN treated cohort with an agreement of 100 out of 104 genes, 85 genes being overexpressed and 15 genes under-expressed, similarly in both groups.

The second cluster of patients was characterized by a lack of the IFN inducible transcriptional response, and was comprised of 38 patients consisting of 8 SPMS IFN treated patients (40 % of SPMS treated cohort), defined as SPMS IFN transcriptional non-responders, and 30 untreated SPMS patients.

To further establish these findings an additional differential expression analysis examining SPMS transcriptional IFN responders (*n* = 12) as compared to SPMS untreated patients (*n* = 30) was performed, and 94 of the 104 DEGs were significantly differentially expressed. In contrast, SPMS transcriptional non-responders (*n* = 8) had no genes at all that were differentially expressed from untreated SPMS patients. To assess if lack of IFN transcriptional response is dependent on the presence of anti-IFN antibodies, IFN NAB status was examined in SPMS IFN treated transcriptional non–responders. In 75 % of these patients IFN NAB were found to be NAB negative.

### Association of IFN inducible transcriptional clusters with clinical patient characteristics

We examined the demographic and clinical characteristics of SPMS patients, which may account for transcriptional differences between: IFN-treated SPMS transcriptional responders, non-responders and untreated SPMS patients (Table 2). Notably, SPMS transcriptional responders had significantly: 1. Shorter disease duration (12.9 ± 2.1 years vs. 19.0 ± 1.5 years in non-responders, *p* = 0.03, and 22.3 ± 2.8 years in untreated SPMS patients, *p* = 0.01); 2. Higher annual EDSS progression rate from disease onset (0.6 ± 0.1 vs. 0.3 ± 0.1, *p* = 0.02 in non-responders); 3. Higher annual relapse rate (0.8 ± 0.1 transcriptional responders vs. 0.4 ± 0.04 non-responders and 0.4 ± 0.04 untreated, *p* = 0.01); 4. Shorter time on IFN treatment (3.5 ± 0.8 years transcriptional responders versus 6.4 ± 1.2 non-responders, *p* = 0.05). These differences reflect that SPMS patients that are transcriptional responders have a more aggressive disease course as compared with transcriptional non-responders.

**Table 2:** Demographical and clinical characteristics of SPMS patients

	Treated IFN transcriptional responders	Treated IFN transcriptional non responders	Untreated
*N*	12	8	30
Age (years)	52.4 ± 2.8	54.9 ± 1.5	51.3 ± 1.4
F (M)	10 (2)	6 (2)	18 (12)
Disease duration (years)	12.9 ± 2.1(*)(**)	22.3 ± 2.8	19.0 ± 1.5
EDSS	6.3 ± 0.2(**)	6.3 ± 0.3	7.2 ± 0.1
Time to EDSS 6 (years)	11.4 ± 2.0	13.5 ± 1.4	12.6 ± 1.3
Annual EDSS change	0.6 ± 0.1(*)	0.3 ± 0.1	0.4 ± 0.1
Relapse rate	0.8 ± 0.1(*)(**)	0.4 ± 0.04	0.4 ± 0.05
Treatment duration (years)	3.5 ± 0.8(*)	6.4 ± 1.2	NA

*p <0.05 between SPMS transcriptional responders vs. non-responders

**p < 0.05 between SPMS transcriptional responders vs. untreated

### Verification of IFN inducible IFI 44 and OAS1 by qRT-PCR

**Fig. 4:**
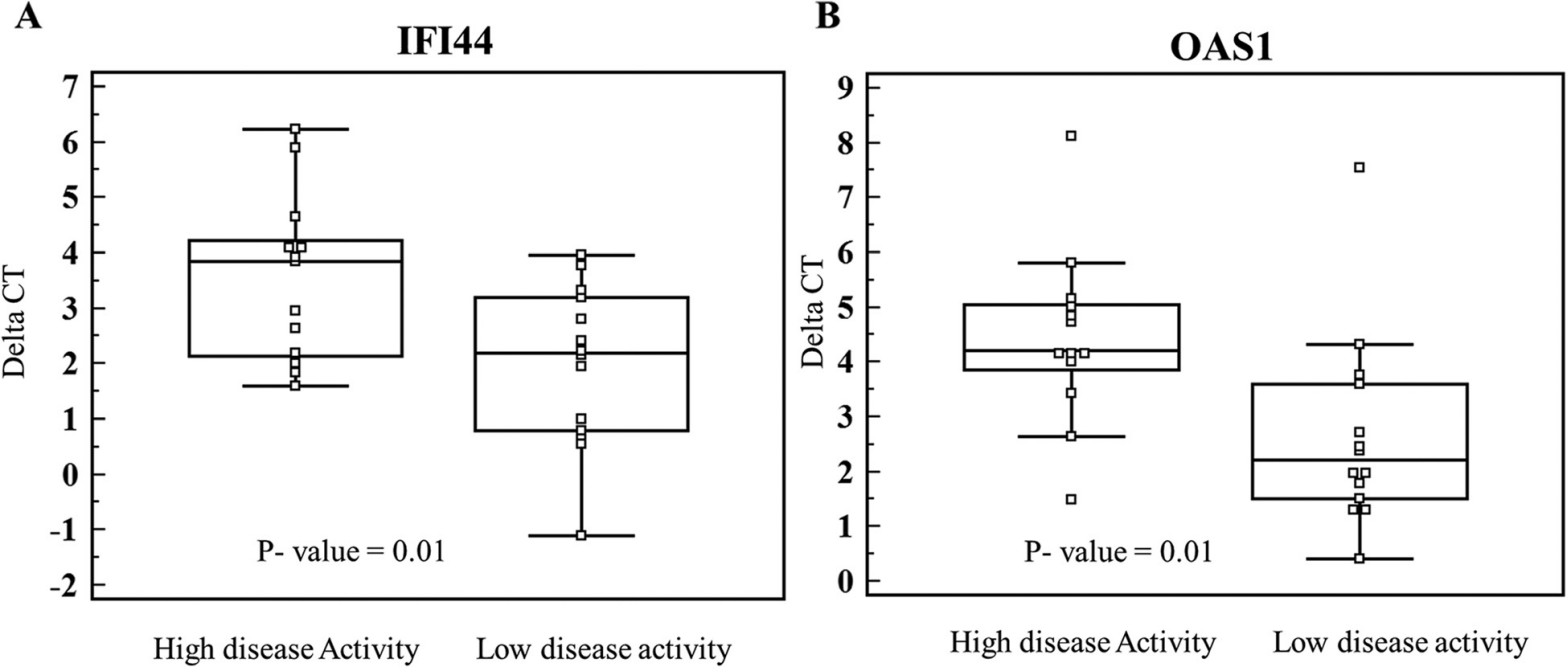
qRT-PCR verification of key IFN inducible genes. Expression of IFN induced (**a**) IFI44 and (**b**) OAS1 in SPMS IFN treated patients

Verification was performed on an additional independent group of 27 IFN treated SPMS patients. We compared 13 patients (age 52.7 ± 5.2 years, 8 females, EDSS 6.3 ± 0.2, disease duration 20.3 ± 2.2 years), characterized by a high disease activity defined as continued disability progression two years following sampling (EDSS increase by 0.7 ± 0.1) with 14 patients (age 58.7 ± 2.3 years, 12 females, EDSS 6.0 ± 0.1, disease duration 22.0 ± 1.9) who had low activity disease, defined as no progression of EDSS two years following sampling. IFN NAB status was similar between groups, each having 4 patients that were positive for IFN NAB. Patients with higher disease activity had significantly higher expression levels of IFN inducible IFI44 (3.5 **±** 0.4 vs. 1.9 **±** 0.4, *p* = 0.01) and OAS1 (4.4 ± 0.4 vs. 2.6 ± 0.5, *p* = 0.01) genes (Fig. 4).

## Discussion

SPMS is a devastating progressive phase of MS, whereby patients gradually develop permanent disability. Clinical response to IFN treatment in SPMS patients is heterogeneous and overall IFN is unable to prevent disease progression. However, it has been reported that a subgroup of SPMS patients with active disease, reflected by short disease duration and high relapse rate prior to transforming to the SP phase, exhibit better therapeutic response to IFN treatment [9]. In the current study, we further expanded this concept by demonstrating that within our cohort of SPMS patients, 60 % characterized by active disease had blood transcriptional signature of response to IFN treatment similar to that of RRMS patients that were good clinical responders to treatment. Our findings, obtained by microarray gene expression technology, were than verified with an independent cohort of patients, by examining expression of key IFN- inducible genes by qRT-PCR.

Although in SPMS the dominant pathological element is neurodegenerative, this is not to say that inflammation is absent. In a large pathological study of the brain in SPMS patients, it was shown that neurodegeneration is associated with ongoing inflammation, and reduction of the inflammatory process correlated to parallel reduction in neurodegeneration [6]. These findings strengthen the biological reasoning that identification of SPMS patients with an active inflammatory component that could be targeted by IFN is important for the selection of appropriate patients for treatment. Indeed, we found a subset of SPMS patients, who have a natural history of disease characterized by a more aggressive disease course even prior to IFN treatment, that exhibit an RRMS like transcriptional response to IFN treatment and therefore have the minimum molecular requirement that can be translated to clinical responsiveness to IFN treatment. In contrast, SPMS patients (IFN transcriptional non responders), who do not exhibit any molecular response to IFN treatment, are less likely to show beneficial clinical response to treatment.

The transcriptional signature we identified in RRMS IFN treatment responders is in agreement with that seen in previous studies [17, 26, 27]. This signature showed high enrichment for IFN signaling and featured genes related to immunoregulatory effects including Jak/STAT signaling, antiviral activity, immune-regulation, cell survival and apoptosis, cell cycle control, and transcription regulation. This signature is known to cause an anti-inflammatory shift in expression of cytokines and chemokines, modulation of T cell adhesion and blood brain barrier extravasation, and an overall reduction in activated T cells entering the brain [15].

The identified IFN transcriptional signature included genes which have previously been shown to correlate with the clinical effects of treatment in RRMS patients. A study examining expression level of MxA, an IFN induced gene, among 126 RRMS patients, showed that patients with lower expression of MxA had a higher rate of relapses [28]. Another study of 77 RRMS patients, found that patients with good clinical response to IFN showed up regulated expression levels of 8 IFN induced genes including IFIT1, IFIT3, IFI44, and OASL after two years of treatment [18]. Two genome wide association studies (GWAS) have been conducted in order to identify genetic determinants of response to IFN beta treatment [29, 30]. These studies discovered a total of 23 significant single nucleotide polymorphisms (SNP) associated with better response to interferon, of which ADAR gene, involved in post transcriptional modification of mRNA and suppression of type 1 interferon signaling, was also found to be differentially expressed in our 104 DEG's signature of IFN inducible profile. Interestingly, ADAR gene was one of the few validated and confirmed SNPs in both of the previous GWAS studies [31]. Our findings provide additional evidence to support the role of ADAR gene on influencing molecular response to IFN.

The relation between expression of IFN inducible genes and clinical response to treatment, as observed in RRMS patients, suggests that in SPMS patients as well, the ability of inflammatory blood cells to have a transcriptional response to IFN, as we observed in 60 % of patients, is a necessary prerequisite for clinical response.

One possible explanation why 40 % of SPMS treated patients in our cohort lack an IFN inducible transcriptional signature is long treatment duration. Previous studies, focusing on long term IFN treatment in RRMS, had shown that patients may cease to express the IFN related expression signature [27]. This is in agreement with our currents findings demonstrating SPMS transcriptional non-responders also had longer treatment duration. Additionally, the production of IFN antibodies could neutralize treatment effect causing a transcriptional switch from IFN inducible to non-inducible signature. However, in our patient cohort this was not the case, with a majority of our SPMS treated transcriptional non responding patients negative IFN antibody status.

## Conclusion

The present study is innovative in demonstrating the concept that there is a subpopulation of IFN treated SPMS patients, who have a transcriptional profile which is similar to that identified in IFN treated clinically responding RRMS patients. We believe this finding may have therapeutic implications that can assist in future tailoring of IFN treatment in SPMS patients.

## Additional files

Additional file 1: Table S1. List of DEGs characterizing RRMS clinical responders. (XLSX 17 kb) Additional file 2: Table S2. List of DEGs characterizing SPMS untreated vs. RRMS untreated patients. (XLSX 21 kb)

## Abbreviations

IFN: Interferon
RRMS: Relapsing remitting multiple sclerosis
SPMS: Secondary progressive multiple sclerosis
IMD: Immunomodulatory therapy
PBMC: Peripheral blood mononuclear cells
EDSS: Extended disability status score
DEG: Differentially expressed genes
NAB: IFN Neutralizing Antibody
qrPCR: Quantitative reverse-transcription-PCR
GWAS: Genome wide association studies
SNP: Single nucleotide polymorphisms
